# Effect of electrochemotherapy on human herpesvirus 8 kinetics in classic Kaposi sarcoma

**DOI:** 10.1186/s13027-017-0147-4

**Published:** 2017-06-19

**Authors:** Noemy Starita, Gianluca Di Monta, Andrea Cerasuolo, Ugo Marone, Anna Maria Anniciello, Gerardo Botti, Luigi Buonaguro, Franco M. Buonaguro, Maria Lina Tornesello

**Affiliations:** 1 Molecular Biology and Viral Oncology Unit, Istituto Nazionale Tumori IRCCS “Fond. G. Pascale”, 80131 Naples, Italy; 2 Department of Surgery “Melanoma, Soft Tissues, Head and Neck, Skin Cancers”, Istituto Nazionale Tumori IRCCS “Fond. G. Pascale”, Naples, Italy; 3 Department of Pathology, Istituto Nazionale Tumori IRCCS “Fond. G. Pascale”, Naples, Italy

**Keywords:** HHV8, Kaposi sarcoma, Electrochemotherapy, Viral load, ORF26, K1

## Abstract

**Background:**

Electrochemotherapy (ECT) has shown to be an effective treatment for cutaneous and subcutaneous Kaposi sarcoma (KS) lesions. However, no study has investigated the impact of ECT treatment on the kinetics of human herpesvirus type 8 (HHV8), which is considered the necessary causal agent of KS. We aimed to evaluate HHV8 viral load and expression levels in patients affected by classic KS who received one or more ECT treatments and have been followed semi annually for up to four years.

**Methods:**

A total of 27 classic KS patients were enrolled in this study. Tumour biopsies and blood samples were obtained before ECT treatment. Additional blood samples were collected at six month intervals for 12–48 months. HHV8 viral load and expression profiles of latent (ORF72 and ORF73) and lytic (K2, K8, K8.1, K10/K10.1, K10.5/K10.6 and ORF16) genes were assessed in all samples by real-time PCR. HHV8 ORF26 and K1 regions were amplified and subjected to direct nucleotide sequencing followed by phylogenetic analysis for variant identification.

**Results:**

All KS biopsies and 46.4% of peripheral blood mononuclear cells (PBMCs) collected before ECT treatment were positive for HHV8 DNA. Viral load ranged from 0.02 to 2.3 copies per cell in KS lesions and 3.0 × 10^−7^ to 6.9 × 10^−4^ copies per cell in PBMCs. Overall, latent ORF72 and ORF73 as well as lytic K2, K8 and K10/K10.1 were expressed in all KS biopsies. ORF16 mRNA was detected in 71.4% and both K8.1 and K10.5/K10.6 mRNAs in 57.1% of KS samples. The ORF72, ORF73 and K2 transcripts were amplified in 37.5%, 25% and 25% of PBMCs collected before ECT, respectively. After the first ECT session, complete response was achieved in 20 out of 27 (74.1%) patients and HHV8 DNA was detected in four out of 27 (14.8%) PBMC samples at six month follow up. Phylogenetic analysis of ORF26 amplimers showed that most viral variants belonged to A/C (82.3%), and few to C2 (5.9%) or C3 (11.8%) subtype. The K1/VR1 variants fell into A (33.3%) and C (66.7%) HHV8 clade. No correlation was found between HHV8 subtypes and ECT complete response.

**Conclusions:**

ECT therapy has a significant effect on HHV8 kinetics in patients with classic KS. The complete remission of patients was accompanied by clearance of circulating virus.

## Background

Kaposi sarcoma (KS) is a locally aggressive vascular tumour generally presenting with cutaneous multiple patches, plaques or nodules [1]. Epidemiological and clinical forms of KS comprise: 1) the classic or sporadic KS, mainly developing in elderly men of Mediterranean and Eastern European origin; 2) the African or endemic KS; 3) the iatrogenic or immunosuppression-associated KS; and 4) the epidemic or human immunodeficiency virus (HIV)/acquired immunodeficiency syndrome (AIDS)-related KS [2, 3].

The frequency of KS has dramatically increased in Europe and United States during AIDS epidemic and remains one of the most frequent tumours among HIV positive patients [4]. The incidence of classic KS has a marked geographical variation between and within European regions with the highest incidence in southern Europe (ASR 0.8/100,000), where it shows a bimodal distribution with peaks in the range of 35–39 years and over 65 [5].

Classic KS is characterized by cutaneous lesions distributed mainly on the lower extremities and trunk and often associated with venous stasis, lymphedema or hyperkeratosis [6, 7]. Different therapeutic options are available depending on disease stage such as lesion excision, laser treatment, cryosurgery, radiotherapy and intra-lesion injection of cytotoxic drugs [8, 9]. More recently electrochemotherapy (ECT) has shown to be an effective local treatment for KS lesions [10]. ECT is a technique combining the high intensity electric pulses with the administration of non permeant or poorly permeant chemotherapeutic agents, such as bleomycin or cisplatin, to facilitate drug delivery into the tumour cells [11, 12]. ECT has shown to be very effective for the local treatment of cutaneous metastatic nodules and primary skin tumours including cutaneous KS [10, 13, 14].

Human herpesvirus type 8 (HHV8) is recognized as the etiological agent of all forms of KS and two lymph proliferative disorders, multicentric Castleman disease and primary effusion lymphoma (PEL) [15–19]. The infection is predominant in men (male to female ratio 10–15:1) and increases with age suggesting that transmission occurs throughout the whole life [20].

 HHV8 infects “spindle” shaped cells, endothelial and epithelial cells as well as B lymphocytes, monocytes and CD34+ stem cells [21–23]. HHV8 encodes several oncogenic viral homologues inducing host cell proliferation, immune evasion and angiogenesis [24]. The viral life cycle comprises the latent and lytic viral programs [25]. The HHV8 genome is maintained in the host cells as an episome and during the latent stage few viral genes are expressed in spindle cells, particularly ORF71 (v-FLIP), ORF72 (v-cyclin) and ORF73 (LANA-1), K12 (kaposin A, B and C), K1 (VIP) and several miRNA encoding genes [26]. The virus undergoes lytic replication in a limited fraction of latently infected cells and expresses many proteins which through autocrine and paracrine mechanisms directly or indirectly cause alteration of several pathways in the surrounding spindle cells [27].

Several studies have evaluated the association between HHV8 viral load in peripheral blood and KS progression in HIV-infected patients [28, 29]. The aim of this study was to evaluate the effect of ECT on HHV8 viral load and the role of HHV8 latent (ORF72 and ORF73) and lytic (K2, K8, K8.1, K10/K10.1, K10.5/K10.6 and ORF16) gene expression in tumour biopsies and peripheral blood mononuclear cells (PBMCs) from patients affected by classic KS with different response to ECT.

## Methods

### Patients and samples

This prospective, single-centre study included 27 consecutive patients diagnosed with classic KS lesions on the inferior limbs (24 males and 3 females) that were treated by ECT at the Istituto Nazionale Tumori “Fondazione G. Pascale” of Naples from February 2009 to November 2013. All KS patients were classified as stage I or II based on objective criteria according to Brambilla staging system [30]. Each patient was asked to give a written informed consent to participate to the study and was invited to fill an epidemiological questionnaire regarding lifestyle, risk factors and anamnestic data. Demographic features including patient origin, age at onset, gender, as well as clinical characteristics such as localization of lesions, treatment modalities, tumour recurrence at the time of observation were also recorded. All patients underwent concurrent incisional biopsy and blood sampling for histological examination and HHV8 molecular analyses at the time of enrolment. Further blood samples were collected every six months during follow up visits after ECT treatments. Each biopsy was divided in two sections, one processed for pathological examination and the second stored in RNAlater stabilizing solution (Ambion®) at −80 °C. All cases included in the study were negative for HIV-1/2 antibodies by macro enzyme immunoassay. All patients underwent tumour staging by lymph node and abdominal ultrasound as well as chest X-ray. This study was approved by the Institutional Scientific Board and by the Ethical Committee of the Istituto Nazionale Tumori “Fond Pascale”, and is in accordance with the principles of the Declaration of Helsinki. All patients provided written informed consent.

### Electrochemotherapy treatment regimen

The ECT treatment regimen was previously described [10]. The treatment was repeated in patients who presented multiple lesions, difficult to treat in a single session or if a complete response was not achieved at a first ECT application. The aim of the treatment was curative and repeated several times until the disappearance of the treated lesions.

### DNA and RNA isolation

PBMCs were isolated from fresh blood by Ficoll-Hypaque™ density gradient. The mononuclear cell layer was resuspended in 500 μl of RNAlater and stored at −80 °C. Genomic DNA was extracted from the PBMCs and KS biopsies according to previously described protocol [31]. In particular, all samples were digested with proteinase K treatment (150 μg/ml) by 2 h incubation at 56 °C or 30 min at 37 °C for biopsies or PBMCs, respectively, in 50–500 μl of lysis buffer (10 mM Tris-HCl, pH 7.6, 5 mM EDTA, 150 mM NaCl, 1% SDS), followed by phenol- chloroform-isoamyl alcohol (25:24:1) extraction and ethanol precipitation in 0.3 M sodium acetate (pH 4.6).

Total RNA was isolated from frozen biopsies and PBMCs using Trizol™ Reagent (Invitrogen™) following the manufacturer’s instructions. Briefly, tissues and PBMCs were resuspended in Trizol reagent and tissues were homogenized by TissueLyser (Qiagen GmbH). After 15 min centrifugation at 13000 rpm, the top phase was collected and total RNA was precipitated with 0.5 volume of isopropyl alcohol 10 min at room temperature. RNA pellet was washed with 75% ethanol, resuspended in sterile water and stored at −80 °C. RNA quality and concentration was analysed with RNA Nano chipassay on Agilent 2100 Bioanalyzer (Agilent Technologies).

### HHV8 viral load

All DNA samples were subjected to HHV8 quantification by real time PCR using primer pairs amplifying a region within the HHV8 ORF26 as previously described [32]. The PCR reactions were performed in a total volume of 25 μL containing 12.5 μL of iQ SYBR Green Supermix (100 mM KCl, 40 mM Tris-HCl, pH 8.4, 0.4 mM of each dNTP and 50 units/mL iTaq DNA polymerase, 6 mM MgCl_2_, 20 nM SYBR Green I [BioRad Laboratories, Inc]), 5 pmol of each primer, and 100–500 ng of genomic DNA. All experiments were performed in the CFX96 Real Time System (BioRad Laboratories, Inc). Dilution series (10 to 10^4^ copies) of genomic DNA extracted from HHV8-infected BCBL-1 cell line (containing 70 copies per cell of HHV8 DNA) were used to construct the standard curve.

### HHV8 latent and lytic gene expression

Total RNA (100 ng) was reverse transcribed into cDNA using iScript™ cDNA Synthesis kit (BioRad Laboratories, Inc.) according to manufacturer’s instructions. The reaction was performed in 20 μl reaction mixture containing 4 μl of 5× iScript Reaction Mix, 1 μl of iScript Reverse Transcriptase and nuclease-free water in a Perkin Elmer thermo cycler (Gene Amp PCR System 2400, Roche Diagnostic System) with the following steps: 5 min at 25 °C, 30 min at 42 °C and 5 min at 85 °C.

A SYBR Green real time PCR method was used to evaluate latent (ORF72 and ORF73) and lytic (K2, K8, K8.1, K10/K10.1, K10.5/K10.6 and ORF16) gene expression in biopsies and PBMCs. Specially, oligoprimers were designed with Beacon Designer software (Premier Biosoft) and used for real time PCR (Table 1). All reactions were performed in the Bio-Rad CFX96 real time PCR Detection System using 1 μl of the cDNA (equivalent to 5 ng RNA), 12.5 μL of iQ SYBR Green supermix (BioRad Laboratories, Inc.), and 5 pmol of each primer in a final volume of 25 μL. Thermal cycling consisted of a denaturation step at 95 °C for 3 min, followed by 40 cycles of 55 °C annealing for 30 s, 72 °C extension for 30 s and 95 °C denaturation for 30 s.

**Table 1:** PCR primer sequences used to amplify HHV8 latent and lytic mRNAs

Locus	Primer name	Sequences (5′ – 3′)	cDNA Amplicon (bp)
Human			
Il-1α	DM151 DM152	GTCTCTGAATCAGAAATCCTTCTATC CATGTCAAATTTCACTGCTTCATCC	421
HHV8 latent			
ORF72	ORF72A ORF72B	CCGCGCTTTTTAACTTCTGACTCT GCTGATAATAGAGGCGGGCAATGA	507
ORF73	ORF73A ORF73B	ACTATGGAAGATTGTAGG TATATGTGTATTGTCAGAAC	106
HHV8 lytic			
K2	ORFK2F ORFK2R	ATTGAGTCTCTGAATGAG TTCAAGTTGTGGTCTATC	155
K8	ORFK8F ORFK8R	AAAGCATACACAAGACAG AAATAATCTGTTCCTTATGTG	100
K8.1	ORFK8.1F ORFK8.1R	CCGATGCCTTAATATCAG TTCCTCTAGTCGTTGTAG	155
K10.5/K10.6	VIRF3F VIRF3R	GAGTTTTCACCCACAAATG CAGGACTCACCTACACAG	107
K10/K10.1	VIRF4F VIRF4R	TGTTCAGTCGTTATATCA TATTCAGTGTCTGTTGTC	182
ORF16	ORF16F ORF16R	TGGCTATACTGACCTTTG GCTTCATACGCATATACAG	78 °C

The amplification of IL-1α was the positive control of reverse transcription. The reaction conditions were the same used for the viral mRNA quantification and the primer sequences are reported in Table 1.

### HHV8 ORF26 and K1 amplification and DNA sequencing

The HHV8 ORF26 region was amplified by seminested PCR using in the outer reaction the oligonucleotide ORF26LR1F1 (5′-GCAGTATCTATCCAAGTA-3′) with ORF26LR1R1 (5′-GGAACCAAGGCTGAT-3′) and in the inner reaction the ORF26LR1F1 with ORF26LR2R2 (5′-ACAGATCGTCAAGCA-3′). The HHV8 K1 region was amplified by seminested PCR using K1LR1F1 (5′-ATCAAGATGTTCCTGTAT-3′) and K1LR1R1 (5′-TATAGTATTTAGTTTGTGACA-3′) in the outer reaction and K1LR1F1 and K1LR2R2 (5′-CATTATTTCCAGAGGTAG-3′) in the inner reaction yielding an amplimer of 250 bp encompassing the hypervariable region VR1 (aa 54–93). The outer PCR amplification reactions were performed in 50 μl reaction mixture containing 300 ng of target DNA, 5 pmol of each primer, 2.5 mM MgCl_2_, 50 nM of each dNTP and 5 μl Hot Master buffer and 2.5 U of Hot Master Taq DNA Polymerase (5 Prime GmbH, Hamburg, Germany) while the inner PCR amplifications were performed in 50 μl reaction mixture containing 5 μl of outer reaction. All samples were amplified in the Agilent Technologies Sure Cycler 8800 thermal cycler with the following steps: an initial 2 min denaturation at 94 °C, followed by 45 amplification cycles for ORF-26 and 38 cycles for ORF-K1 of 55 °C and 52 °C respectively for 45 s, 68 °C for 1 min, 94 °C for 15 s and a 5 min final elongation at 68 °C. Reaction mixture containing genomic DNA, extracted from NIH 3 T3 murine cell line, was used as negative control and included in every set of 5 clinical specimens.

All HHV8 amplimers were subjected to bidirectional direct sequencing analysis by Eurofins Genomics Srl. Nucleotide sequences were edited with Chromas Lite 2.01 (http://www.technelysium.com.au/chromas.html) and converted to FASTA format.

### Phylogenetic analysis

Multiple alignments of HHV8 nucleotide sequences from the present study and reference strains reported in the GenBank were performed with clustal W tool of MegAlign program of the Lasergene software (DNASTAR Inc., V7.0.0). All reference sequences were downloaded from the National Centre for Biotechnology Information (https://www.ncbi.nlm.nih.gov/nuccore/?term=HHV-8). Reference sequences for each HHV8 ORF26 subtype were DQ984689.1 (BCBLR, A/C), DQ984768.1 (HKS15, R), DQ984785.1 (431 K, B1), DQ984789.1 (021 K, B2), and DQ984759.1 (HKS21, J); whereas reference sequences for each HHV8 K1 subtype were AF133038 (BCBL-R, A1), AF133039 (BCBL-B, A4), JN800486.1 (QLD-KS-8, A), AF151688.1 (US3/ts55, A), JN800487.1 (QLD-KS-9, A), AF130284.1 (Ife5, A), KF781665.1 (1ZA, A), GU097427.1 (KE-231, A),; AF133041 (ARM72, C1), AF133042 (BC2, C3), FJ866517.1 (TYKS12, C), DQ394064.1 (I10, C), DQ394068.1 (N1, C), DQ394038.1 (D4.2, C) and DQ394054.1 (D18, C).

Phylogenetic trees were constructed transforming the aligned sequence data into a distance matrix by the Kimura’s two parameter model (Kimura, 1980), followed by the neighbor-joining bootstrap analysis [33], which was executed with the MEGA software (version 6.0) [34]. Boot strapping, with 1000 replicates confirmed the robustness of the three major branches with bootstrap values above 90%.

### Statistical analysis

The statistical analysis was performed using Graph Pad Prism Software version 6.00. Two-tailed Fisher’s exact test were used for comparison of categorical data. U Mann–Whitney test were used to evaluate differences in viral load. Differences were considered to be statistically significant when *P* values were less than 0.05.

## Results

This study included a total of 27 patients (24 males and 3 females) affected by classic KS, with a median age at the diagnosis of 74 years (range 43–88 years of age) (Table 2). The majority of patients (74.1%) were above 65 years while 25.9% were below 65 years of age. All patients received single or multiple ECT treatments as previously described [10], and after 24 months a clinical response was obtained in all of them according to RECIST guidelines. In particular, a complete response was observed in 20 out of 27 patients (74.1%) after a single ECT session, in three patients (11.1%) after two treatments and four patients (14.9%) after three to six ECT applications. The mean interval between two-consecutive treatments was 145 days. The clinical response to ECT was evaluated four weeks after ECT treatment and monitored every three months during a follow-up period ranging between 24 and 68 months.

**Table 2:** Patients characteristics, KS lesion sites and number of ECT treatments for each patient

ID Patient	Years of diagnosis	Sex	Age	Localization	N° ECT treatment	Response
KS288	2009	M	45	Lower limb bilateral	2	CR
KS290	2009	M	85	Left lower limb	1	CR
KS289	2010	M	73	Right foot	3	CR
KS293	2010	M	74	Right lower limb	2	CR
KS294	2010	M	79	Left upper limb	1	CR
KS295	2010	F	69	Right lower limb	1	CR
KS296	2011	M	77	Right lower limb	6	CR
KS298	2011	M	66	Right foot	1	CR
KS299	2011	F	86	Right foot	1	CR
KS300	2011	M	85	Right foot	5	CR
KS301	2011	M	63	Right foot	1	CR
KS302	2011	M	77	Right lower limb	1	CR
KS307	2012	M	72	Right foot	1	CR
KS309	2012	M	88	Right lower limb	1	CR
KS310	2012	M	88	Right foot	1	CR
KS315	2012	M	70	Left lower limb	1	CR
KS339	2012	M	78	Left lower limb	1	CR
KS319	2013	F	88	Right lower limb	1	CR
KS327	2013	M	73	Right foot	1	CR
KS329	2013	M	79	Right lower limb	1	CR
KS330	2013	M	83	Left foot	1	CR
KS332	2013	M	46	Right lower limb	1	CR
KS333	2013	M	83	Right lower limb	2	CR
KS334	2013	M	46	Left foot	1	CR
KS335	2013	M	72	Right foot	1	CR
KS340	2013	M	43	Right foot	4	CR
KS341	2013	M	55	Left foot	1	CR

*CR* complete response

**Fig. 1:**
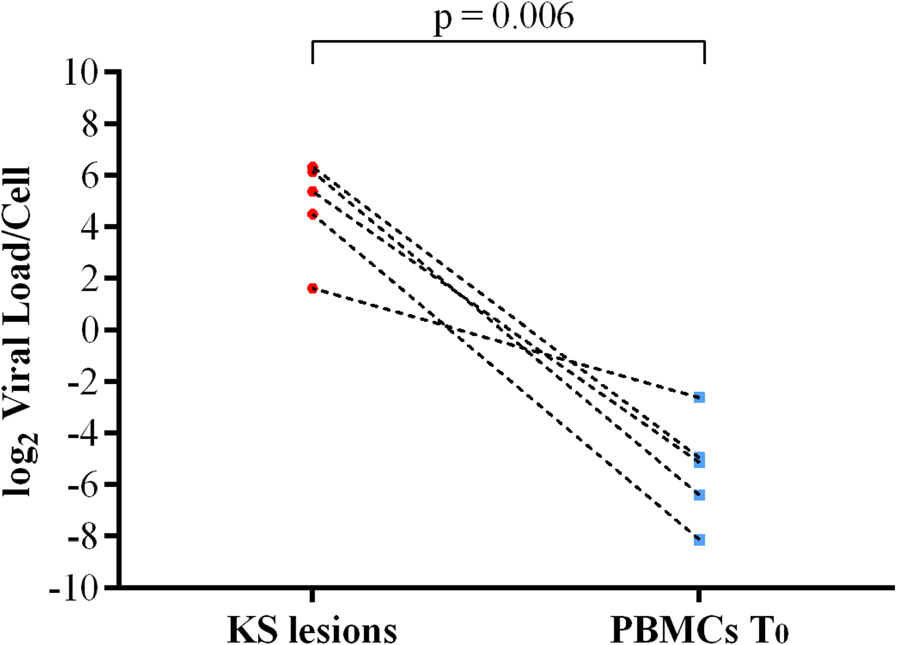
HHV8 viral load in KS lesions and PBMCs before ECT treatment

**Fig. 2:**
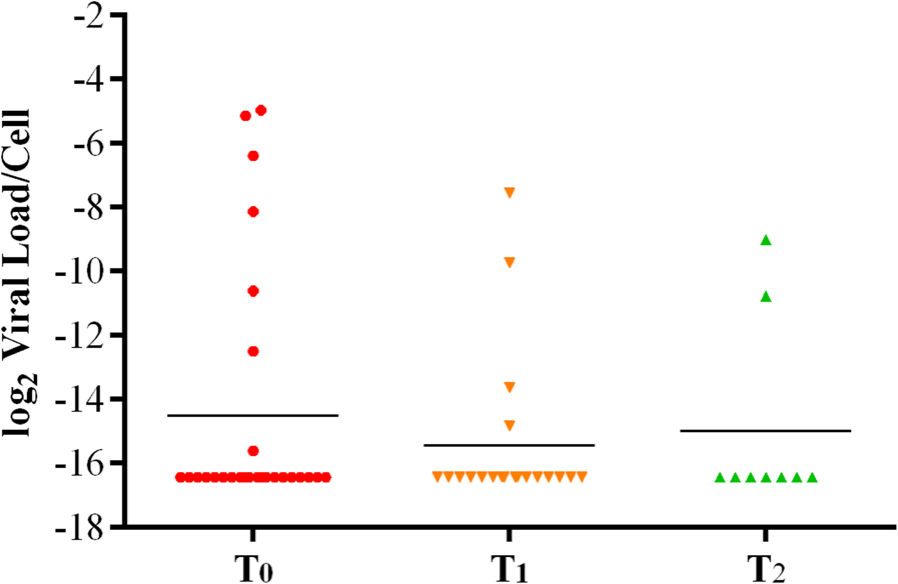
HHV8 viral load evaluated at different time intervals following ECT

KS biopsies were available from 16 patients. PBMC samples collected at the time of enrolment (T_0_), after approximately 6 months (T_1_) and 12 months (T_2_) after ECT therapy were available for 27, 20 and 9 patients, respectively. HHV8 sequences have been identified in 100% of KS biopsies, in 13 out of 27 (48.1%) PBMC samples at T_0_, in 4 out of 20 (20%) at T_1_ and in 2 out of 9 (22%) at T_2_. (Table 3). HHV8 viral load ranged from 1 to 81 copies per cell in KS biopsies and from 1 × 10^−5^ to 1.6 × 10^−1^ copies per cell in PBMCs at the time of enrolment (T_0_). The differences in viral load between KS lesions and PBMCs were statistically significant (*p* = 0.006), Fig. 1. Following ECT treatments the viral load progressively decreased in PBMCs and became undetectable after 24 months in 11 out of 12 (91.7%) PBMC samples which were HHV8 positive at T_0_, Fig. 2. At the end of the follow-up the single patient who received six ECT treatments reached a complete clinical response but remained positive for HHV8 DNA in PBMCs.

**Table 3:** Frequency of HHV8 DNA in classic KS biopsies and persistence of the virus in PBMCs collected from patients at different times following ECT treatment

	T_0_	T_1_	T_2_	*P* value	
	*N =* 27 (%)	*N =* 20 (%)	*N =* 9 (%)	*T*_*0*_ vs *T*_*1*_	*T*_*0*_ vs *T*_*2*_
Sex				0.25	0.56
M	24 (88.9)	20 (100.0)	9 (100.0)		
F	3 (11.1)	0(0.0)	0 (0.0)		
Age				0.72	1.00
≤ 65 years	6 (22.2)	5 (25.0)	2 (22.2)		
> 65 years	21 (77.8)	15 (75.0)	7 (77.8)		
HHV8 DNA in PBMCs					
Positive	13 (48.1)	4 (20.0)	2 (22.2)	0.07	0.25
Negative	14 (51.9)	16 (80.0)	7 (77.8)		
KS lesion persistence					
Yes	27 (100.0)	8 (40.0)	4 (44.4)	0.0001	0.0003
No	0 (0.0)	12 (60.0)	5 (55.6)		
HHV8 DNA in classic KS					
Positive	27 (100.0)				
Negative	0 (0.0)

The HHV8 expression profiles showed that latent genes ORF72 and ORF73 as well as lytic genes K2, K8 and K10/K10.1 were expressed in all analysed classic KS lesions. The ORF16 transcript was detected in 71.4% and both the lytic K8.1 and K10.5/K10.6 in 57.1% of KS tumours. The ORF72, ORF73 and K2 mRNAs were amplified in 37.5%, 25% and 25% of PBMCs, respectively, collected before ECT treatment. The other HHV8 transcripts were undetectable in all PBMCs. In conclusion, latent and lytic transcripts are frequently expressed in biopsies but occasionally in PBMCs.

The HHV8 ORF26 sequences amplified in 16 KS were subjected to nucleotide sequence and phylogenetic analyses and classified as A/C (13 out of 16, 81.3%), C2 (1 out of 16, 6.3%) and C3 (2 out of 16, 12.5%) subtypes, following the nomenclature proposed by Zong et al. [35].

**Fig. 3:**
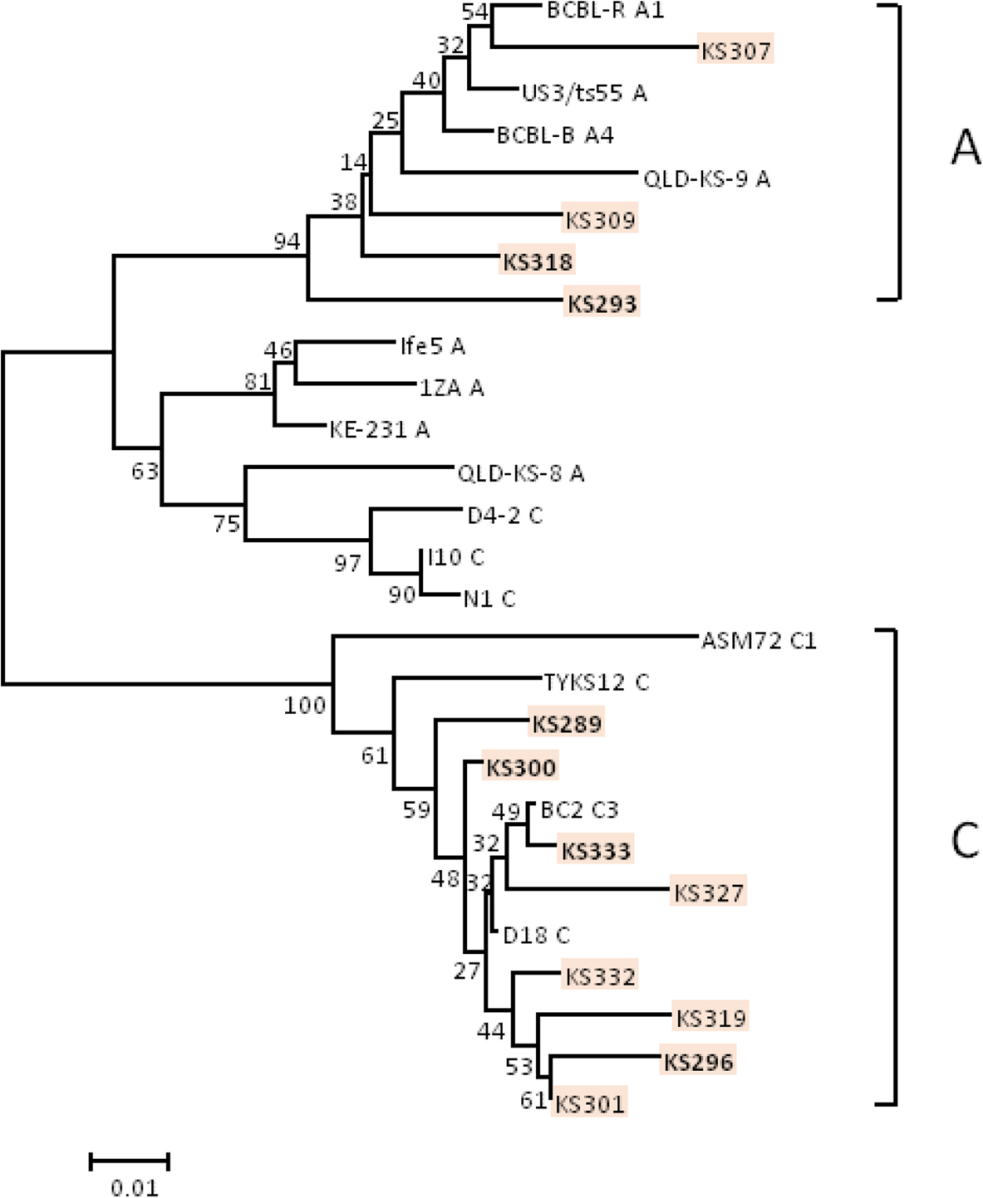
Phylogenetic tree of HHV8 K1 sequences. Numbers above the branches indicate the bootstrap values (1000 replicates) that are greater than 90%

**Fig. 4:**
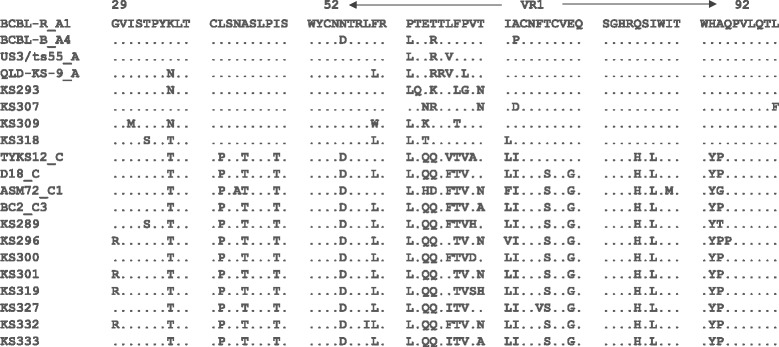
Alignment of amino acid sequences of the K1 VR1 region with GenBank reference strains. The BCBL-R amino acid sequence was used as a consensus sequence in the alignments. *Dots* (*·*) indicate consensus amino acid sequence

The HHV8 K1 amplimers obtained in 12 tumour samples were subjected to phylogenetic analysis showing a nucleotide divergence among them of 0.7–12.3% corresponding to a 4.9–43.3% amino acid divergence in the VR1 (aa 52–92) sequence. Phylogenetic analysis based on the comparison of newly identified K1 sequences with K1 reference strains available in GenBank showed that four out of 12 (33.3%) HHV8 K1 variants belonged to subtype A and eight (66.7%) to subtype C (Figs 3 and 4). Amino acid sequence divergence between subtypes A and C (7.58% to 23.31%) was higher than that observed within subtypes A (3.25 to 9.11%) or C (0 to 13.44%). There was no difference in the response to ECT treatment in patients infected by different HHV8 variants.

## Discussion

The efficacy of ECT for the treatment of KS was first described by Heller et al. in [36]. To date several reports have confirmed the usefulness of ECT on different neoplastic cutaneous lesions including KS [14]. Di Monta et al. [10] proposed ECT as the “new standard of care” in first line treatment of stage I and II classic KS for the high rate of complete response achieved after a single ECT treatment (73.6%). The present study represents an extension of the previous patients cohort and confirms that ECT induces a complete response in above 70% of KS patients after a single session, regardless of tumour size. Moreover we have evaluated HHV8 kinetics in KS patients treated by ECT and we have observed a viral load decline in those patients with a complete clinical response.

The immunological effect of ECT has been first described in mouse models in which numerous mononuclear cells have shown to accumulate in ECT-treated metastatic lesions [37]. Calvet et al., hypothesized that the residual tumour cells not directly affected by ECT treatment, due to insufficient permeabilization or paucity of bleomycin molecules, are secondarily killed by cytotoxic immune cells activated by immunogenic cell death processes in murine colon cancer cells [38]. More recently Di Gennaro et al. analyzed the immune cell profile in biopsies from melanoma patients before and after ECT treatment and reported a relevant local immune response, with decreasing CD4 + FOXP3+ T reg cells and recruitment of CD3 + CD8+ T cells in the treated lesions [39]. It remains unclear whether ECT treatment is sufficient to stimulate a systemic immune response in ECT-treated melanoma patients. Gualdi et al. first reported that ECT has a positive effect on the disappearance of HHV8-positive cells at treated sites 2 months after treatment [40]. In our study we observed a progressive decrease of HHV8 viral load in PBMCs of patients presenting a complete clinical response after a single ECT session suggesting a significant effect of ECT on the activation of the immune system against viral antigens. Only a single patient with recurrent KS lesions had invariable viral load after multiple ECT treatments.

The HHV8 genome encodes for several viral IFN-regulatory factors (vIRFs) differentially expressed in different cell types. In particular, the K9 (vIRF-1) gene is generally co-expressed with ORF73 (LANA-1) in KS cells, while K10.5/K10.6 (vIRF-3 also termed LANA-2) is mainly transcribed in HHV8-associated lymphomas. Such data suggest that different vIRFs encoding genes are important for HHV8 life cycle and that their expression is regulated by cell specific factors [41]. Hosseinipour et al. analysed the expression profile of HHV8 ORFs in 70 KS biopsies and identified a group of tumours with extensive expression of all viral genes (“extended” viral expression), and a group of KS cases with limited gene transcription restricted to the latency genes (“restricted” viral expression) [42]. Such data suggested the existence of multiple molecular subtypes of KS with different sensitivity to treatments [42]. We have analysed the expression profile of latent genes (ORF72 and ORF73) and lytic genes (K2, K8, K8.1, K10/K10.1, K10.5/K10.6 and ORF16) and found them all expressed in 57.1% of KS lesions while the remaining 42.9% lacked the expression of K8.1 and K10.5/K10.6. No difference was observed in clinical response to ECT between the two groups of tumours.

In recent years, much attention has been paid to the association between different subtypes of HHV8 and KS aggressiveness [43, 44]. In our study we have identified A/C, C2 and C3 clades on the basis of ORF26 as well as A and C variants on the basis of K1/VR1 nucleotide sequencing analysis. There was no significant difference between HHV8 subtype distribution, viral persistence and clinical response to ECT treatment.

## Conclusion

This study has shown that ECT treatment of patients with classic KS causes a decline of HHV8 virus load in peripheral blood. The complete remission of KS patients treated with ECT is accompanied in the majority of cases by clearance of the virus in the PBMCs. ECT is very effective in the treatment of classic KS independently from HHV8 viral load, viral gene expression and HHV8 variants.

## Abbreviations

ECT: Electrochemotherapy
HHV8: Human herpesvirus type 8
KS: Kaposi sarcoma
PBMCs: Peripheral blood mononuclear cells
